# A population-based statistical approach identifies parameters characteristic of human microRNA-mRNA interactions

**DOI:** 10.1186/1471-2105-5-139

**Published:** 2004-09-28

**Authors:** Neil R Smalheiser, Vetle I Torvik

**Affiliations:** 1 University of Illinois at Chicago, UIC Psychiatric Institute, MC 912, 1601 W. Taylor Street, room 285 Chicago, IL 60612 USA

## Abstract

**Background:**

MicroRNAs are ~17–24 nt. noncoding RNAs found in all eukaryotes that degrade messenger RNAs via RNA interference (if they bind in a perfect or near-perfect complementarity to the target mRNA), or arrest translation (if the binding is imperfect). Several microRNA targets have been identified in lower organisms, but only one mammalian microRNA target has yet been validated experimentally.

**Results:**

We carried out a population-wide statistical analysis of how human microRNAs interact complementarily with human mRNAs, looking for characteristics that differ significantly as compared with scrambled control sequences. These characteristics were used to identify a set of 71 outlier mRNAs unlikely to have been hit by chance.

Unlike the case in *C. elegans *and *Drosophila*, many human microRNAs exhibited long exact matches (10 or more bases in a row), up to and including perfect target complementarity. Human microRNAs hit outlier mRNAs within the protein coding region about 2/3 of the time. And, the stretches of perfect complementarity within microRNA hits onto outlier mRNAs were not biased near the 5'-end of the microRNA. In several cases, an individual microRNA hit multiple mRNAs that belonged to the same functional class.

**Conclusions:**

The analysis supports the notion that sequence complementarity is the basis by which microRNAs recognize their biological targets, but raises the possibility that human microRNA-mRNA target interactions follow different rules than have been previously characterized in *Drosophila *and *C. elegans*.

## Background

MicroRNAs (miRNAs) are small, ~18–24 nt. noncoding RNAs that are found in all eukaryotes and are cleaved from larger ~70 nt. precursors via the action of Dicer enzyme [reviews: ref. [1,2]]. MicroRNAs are thought to degrade messenger RNAs via eliciting mRNA degradation (if they bind in a perfect or near-perfect complementarity to the target mRNA), or to arrest translation of the mRNAs (if the binding complementarity is imperfect). Although a number of microRNA targets have been identified in plants, *C. elegans *and *Drosophila *[1,2], only one mammalian microRNA target has yet been validated [3,4].

Five different papers have recently appeared that used computational approaches to predict microRNA targets in *Drosophila *[5-7], and mammals [8,9]. These studies only considered hits occurring within 3'-UTR regions that were conserved across related species, and favored or required a short region of perfect complementarity towards the 5'-end of microRNAs. However, there is reason to suspect that the rules governing microRNA-target interactions may not be universal. For example, in plants, most of the known microRNAs bind in a perfect or near-perfect manner to mRNA targets located within the protein coding region (cds) [10,11]. In contrast, in *C. elegans *[12] and *Drosophila *[13], known microRNAs lack long stretches (>10) of complementarity with their targets and generally interact within the 3'-untranslated region (3'-UTR). Furthermore, whereas the 5'-ends of many *Drosophila *microRNAs recognize 5–6 nt. common motifs within the target, these motifs are not a general feature of mammalian microRNAs [14]. Thus, it is conceivable that human microRNA targets do not follow the same constraints as observed in *C. elegans *and *Drosophila*.

In the present paper, we have performed an unbiased statistical analysis of the manner in which human microRNAs interact complementarily with human mRNAs present in the NCBI human RefSeq database, looking for characteristics that differ significantly as compared with scrambled versions of the same microRNA sequences. The results demonstrate several novel features of human microRNA-mRNA interactions that differ from *C. elegans *and *Drosophila*, and identify a short-list of promising candidate microRNA-mRNA target pairs that are unlikely to have arisen by chance.

## Results

Population-wide statistical analyses were first carried out by examining the types of complementary interactions that occur between the set of microRNAs listed in Lagos-Quintana et al [15], and the set of human RefSeq mRNAs downloaded in August 2003. To obtain a fuller list of outlier mRNAs, analyses were repeated using all human microRNAs listed on the Sanger microRNA repository [16] and the set of human RefSeq mRNAs listed as of December 2003 [17].

To define the types of interactions that can occur by chance, ten independent sets ("replications") of scrambled microRNA counterpart sequences were generated and examined for complementarity with the mRNA population. Our underlying assumption is that scrambled sequences will hit mRNA at random and define the "noise" level in any given situation, whereas microRNA sequences will hit the same number of "noise" interactions plus any true targets. Unless otherwise noted, the scrambled sequences were random permutations of the microRNA sequences, keeping constant the overall nucleotide composition. Because microRNAs have a distinctive nonrandom di-nucleotide composition, we also confirmed that key findings were obtained when using scrambled sequences that had similar di-nucleotide composition to the microRNAs.

### 1. Human microRNAs tend to have longer exact hits upon mRNAs than do their scrambled counterparts

**Figure 1:**
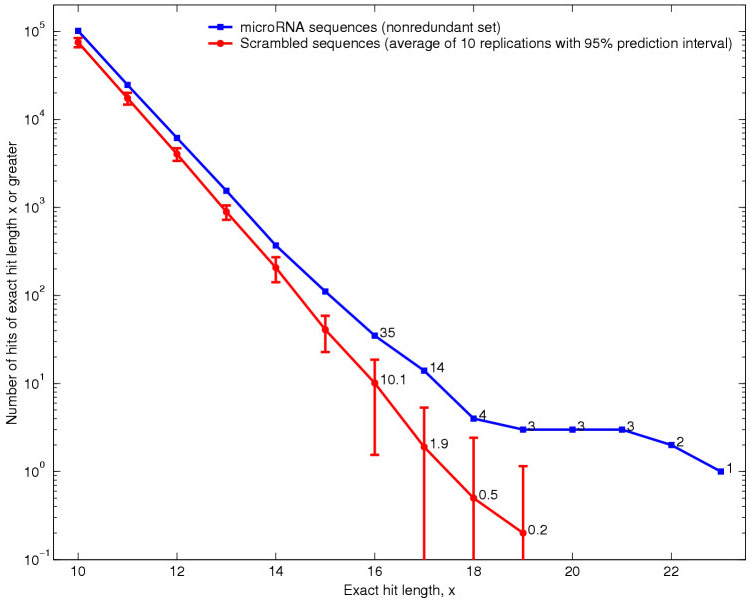
**microRNAs and their scrambled counterparts interact differently with the population of human mRNAs. **Shown are all exact hits ≥ 10 bases long (not counting G:U matches) produced on human RefSeq mRNAs by the set of nonredundant microRNAs, vs. the average of 10 replications of scrambled control sequences. Shown is the number of hits as a function of exact hit length. Only the longest hit was counted: e.g., for a hit of length 18, the two subsets of length 17 in the same hit position were not counted.

First, we characterized the length distribution of exact complementarity between the population of mRNAs vs. the set of nonredundant microRNAs (i.e. those that overlapped by 10 or more bases were collected into groups and the longest member of the group was chosen as nonredundant). MicroRNAs produced significantly longer exact "hits" on mRNAs than their scrambled counterparts when G:U matches were excluded (fig. 1). There was an excess number of hits in the microRNA set relative to scrambled control sequences at all exact hit lengths (10 or greater), and the difference became proportionately greater at longer hit lengths (see below). When microRNAs were compared to scrambled sequences that matched the di-nucleotide composition of microRNAs, similar results were obtained. In contrast, this trend was not observed when G:U matches were included (not shown). Experimental studies suggest that RNA interference and arrested translation can still be elicited when small RNAs are modified to replace a number of Watson-Crick base pairs by G:U matches [18-20]. On the other hand, G:U matches have distinctive binding energy and spatial orientation [21]. Unless otherwise qualified, "exact hits" will refer to complementarity without G:U matches.

### 2. Constructing an outlier set of microRNAs based on cut-offs of exact hit length, gapped BLAST score and presence of multiple hits

As shown in figure 1, there are a total of 101, 279 microRNA hits upon RefSeq sequences hitting exactly ≥10 bases in a row, compared to 75,031 hits produced by scrambled microRNA sequences. The difference (26, 248 hits, distributed among 8258 mRNA sequences) is highly significant (p = 3 × 10^-9^) and suggests that about 1/4 of the total hits in this "10+ set" occur upon "true" biological mRNA targets. Our approach is to identify further the mRNAs that represent statistical outliers (i.e. that are unlikely to be hit by chance) within this larger "10+ set" by comparing properties of hits made by the set of microRNA sequences vs. the set of scrambled sequences. At any given parametric value, the number of hits observed in the microRNA set, minus the number of hits in the scrambled set, provides an estimate of the number of true microRNA targets that satisfy that parametric value. We examined three different hit properties – a) exact hit length, b) gapped BLAST score and c) presence of multiple hits – both alone and combined with each other. Starting from the "10+ set" estimated to contain only 26% true targets (see above), we added additional criteria to compile a list of candidates estimated to contain over 80% true targets.

#### a) Exact hit length

The most important single parameter for discriminating hits produced by microRNAs vs. scrambled sequences appears to be exact hit length. At a cut-off of 17 exact hit length, there were 14 mRNAs hit by the microRNA set that satisfied this criterion, vs. an average of 1.9 mRNAs hit by each of the scrambled sequence sets (fig. 1). Stated another way, this criterion gives a discrimination ratio of 7.4 to 1. A similar discrimination ratio was observed when comparing scrambled sequences maintaining the same di-nucleotide composition as the microRNAs.

#### b) Gapped-BLAST score

**Figure 2:**
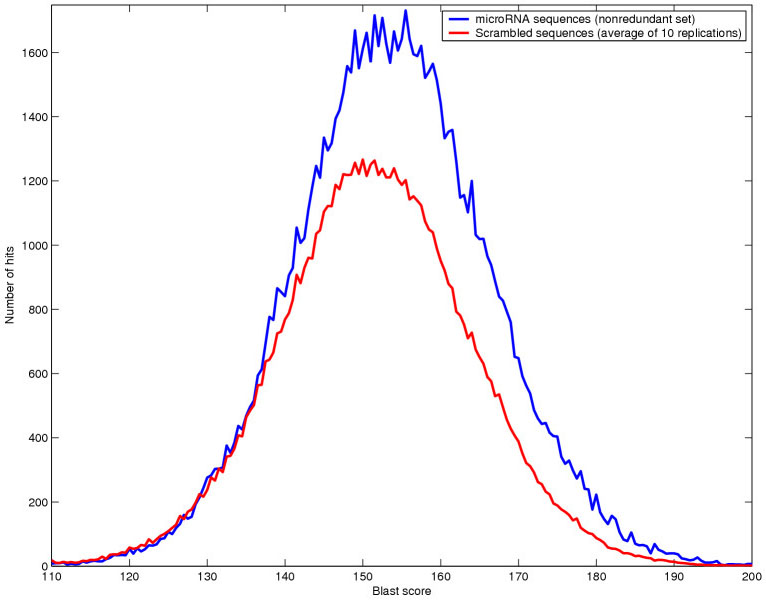
**Distribution of gapped-BLAST scores in hits made by microRNAs and scrambled counterparts. **Without permitting G:U matches in the extension phase, the microRNAs had better average gapped-BLAST scores than scrambled counterparts across all mRNAs in the "10+ set" (153.00 ± 0.03 vs. 150.98 ± 0.01, mean ± s.e.m., p < 0.0001). With permitting G:U matches in the extension phase, the microRNA set showed significantly fewer G:U matches overall relative to scrambled counterparts, even when holding constant the length of the exact hit (2.891 ± 0.004 vs. 2.939 ± 0.001, p < 0.0001).

Overall complementarity of the microRNA-mRNA alignment was also examined within the "10+ set" of individual mRNAs exhibiting exact microRNA hits of at least 10 bases in a row. A modified gapped-BLAST algorithm [22] was used to compute the optimal alignment, employing a weighted score that takes gaps and mismatches into account (r = 10, q = -2.5, G = 8, E = 0.5). Although the two curves overlap quite a bit, their means are significantly different from each other (p < 0.0001), and the microRNA distribution exhibits a discrete "tail" at higher scores that differs significantly from the scrambled distribution (fig. 2).

#### c) Multiple hits

**Figure 3:**
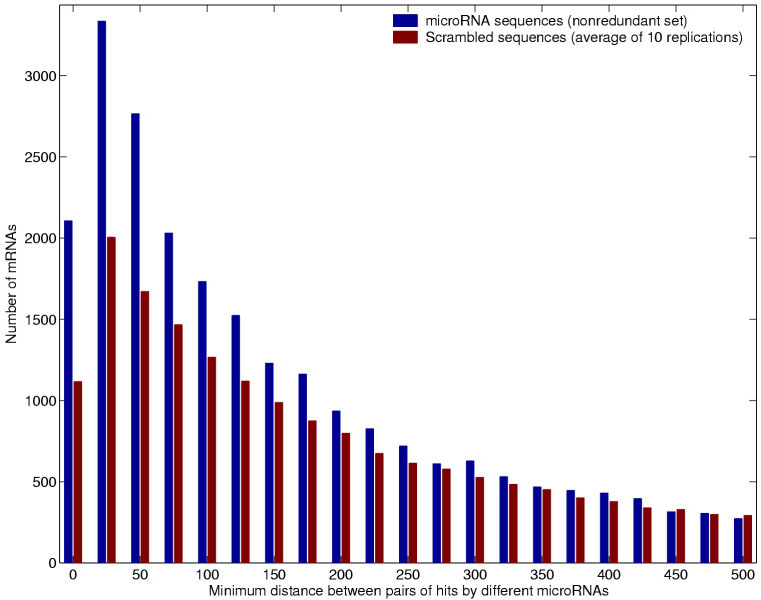
**Number of distinct mRNA sequences which received hits from two or more distinct microRNAs, as a function of the minimum distance between hits. **Distance of 0 or 1 was excluded because this might be produced by partial overlap of microRNA sequences.

In lower organisms, individual validated microRNA targets tend to receive multiple hits by distinct microRNAs [1,2]. mRNA sequences within the "10+ set" were hit by multiple nonredundant microRNAs more often than by their scrambled counterparts, and this was particularly striking when the hits were located close together (fig. 3).

#### d) Combining parameters

When combined, all three parameters (exact hit length, gapped-BLAST scores and multiple hits) gave better discrimination power than using any single feature, supporting the idea that they are relevant to identifying biologically relevant mRNA targets. We examined three different combinations of parameter cut-off values: 1) One combination consisted of targets with multiple hits from distinct microRNAs less than 25 bases apart, with at least one exact hit ≥13 bases and with at least one gapped BLAST score ≥185 (not counting G:U). For the next two lists, we scored only exact hits ≥10 bases long and that occurred ≤50 times within the entire mRNA population; this minimized "noise" arising from common or low-complexity target sequences, albeit at the cost of removing some target sequences that are shared within protein families. 2) Criteria required two or more hits from distinct microRNAs ≤100 bases apart, at least one exact hit ≥14 bases and one gapped-BLAST score of ≥190 (not counting G:U). 3) This required hits ≤500 bases apart, at least one exact hit ≥14 bases, and at least one gapped-BLAST score > 89% of the best-possible score including G:U matches (this takes into account the fact that longer microRNAs have greater possible absolute scores than shorter microRNAs).

All three approaches produced lists of outlier mRNAs that had overlapping members, shared similar characteristics and exhibited similar discrimination ratios. For simplicity and robustness, these have been combined (together with the candidates identified by exact hit length alone) into a single list consisting of 71 outlier mRNAs (Table 1). The combined list was hit by almost the entire set of nonredundant microRNAs (i.e., 107 out of 109). In contrast, scrambled counterpart sequences hit an average of 13.7 ± 1.15 targets and were represented by 54.3 ± 3.5 nonredundant sequences. The combined outlier set gives an overall discrimination ratio of 5.2 to 1, meaning that 57 of the 71 mRNAs are in excess of the number that could be reasonably expected by chance, hence should be accurately assigned as true targets for one or more microRNAs. See  for additional data files including a fully annotated outlier mRNA set, a list of all microRNA hits upon this set (extended with and without including G:U matches), and a list of the nonredundant microRNAs together with their putative mRNA targets.

### 4. Characterizing the mRNA outlier set

**Figure 4:**
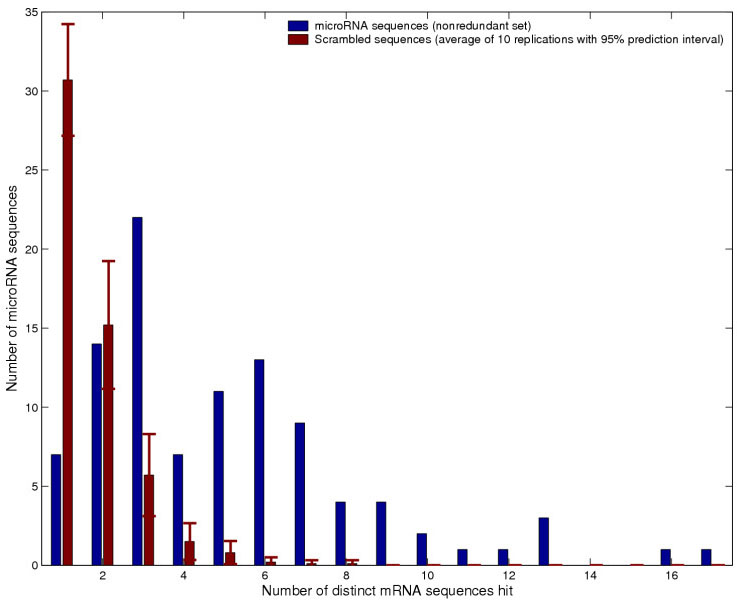
Individual microRNAs hit multiple targets on the candidate list, more often than expected by chance.

The 71 mRNAs in the outlier set had a larger number of microRNA hits per kilobase of target sequence than did the scrambled sequences (2.18 ± 0.1 vs. 1.83 ± 0.085, p = 0.006). As well, individual microRNAs hit multiple (up to 17) distinct members of the outlier set, which again happened significantly more often than by chance (fig. 4). These findings indicate that the outlier mRNAs are different as a whole from the mRNAs that were hit by scrambled counterparts, even those that satisfied the same cut-off criteria.

The outlier mRNA set contained very similar types of targets as predicted by previous computational studies [5-8], including members of the same gene families. For example, Lewis et al. [8] described E2F1 as a candidate target whereas we found E2F6 (Table 1). Transcription factors (including homeobox genes) and nucleic acid-binding proteins are among the top predicted microRNA targets. As well, many other functional categories are represented including kinases, receptors and other signal transduction proteins, membrane and cytokeletal proteins, and effectors of differentiation (Table 1). However, surprisingly, we found that the human candidate microRNA target list also had some features that differed significantly from the known targets in *C. elegans *and *Drosophila*. For example, there was no preference for microRNA hits to be located within 3'-untranslated regions: 5% of hits were located in the 5'-UTR, 1% at the 5'-UTR/coding junction, 67% in the protein coding region, 1% at the coding/3'-UTR junction, and only 26% in the 3'-UTR. This distribution was not significantly different from hits produced by the scrambled sequences. As well, the best microRNA hits upon candidate mRNA targets did not have relatively better target complementarity near their 5'-end: Only 13% of hits had ≥ 7 exact hit length starting at position 1 or 2 relative to the 5'end of the microRNA (vs. 17.5% of hits produced by scrambled sequences).

MicroRNA 145 is particularly interesting because it hits 17 distinct targets on the candidate list, of which a disproportionate number (6) are in the signal transduction category and three of these are related to GTPase activation (Rho GTPase-activating protein (RICS), G protein gamma 7, and hypothetical protein FLJ32810 – containing RhoGAP and SH3 domains; Table 1). A recent study showing that miR-143 and miR-145 are both underexpressed in colorectal neoplasia [23] had previously proposed the first two of these candidates as potential targets. Interestingly, the third target found here is not only novel (XM_350859, RhoGAP-like) but is hit by both miR-143 and miR-145 in close proximity (see additional data file 2 in ), further suggesting that this is likely to be a true biological target for microRNA regulation.

## Discussion

By comparing how the population of microRNAs vs. their scrambled counterparts interact with the population of human RefSeq mRNA sequences, we estimate that the probability of detecting a true microRNA target increases a) as the length of exact complementarity of a "hit" between microRNA and target increases, b) as the overall complementarity of a "hit" increases (allowing for gaps, mismatches and G:U matches), and c) as two or more distinct microRNAs hit the same mRNA in closer proximity. Targets in the outlier mRNA set also received more hits per unit length and more multiple hits from distinct microRNAs than expected by chance. Finally, we found cases in which an individual microRNA hit multiple mRNAs that belonged to the same functional class. The analysis suggests that target complementarity is a major factor in identifying biologically relevant mRNA targets: As values of each parameter increase, the difference between the number of hits in the microRNA set vs. the scrambled set increases steadily, and combining all three parameters gives better discrimination power than using any single feature.

So far, these conclusions agree with five different papers that used computational approaches to predict microRNA targets in *Drosophila *[5-7], and mammals [8,9], using different strategies, criteria and filters than employed here. However, three significant differences were observed between human mRNAs in the outlier set and *Drosophila *targets: 1) Human microRNAs hit mRNAs with exact hit lengths extending much longer than observed in *Drosophila*, up to and including perfect complementarity. 2) Human microRNAs hit candidate mRNA targets within the protein coding region about 2/3 of the time. (This resembles the manner in which plant microRNAs hit their mRNA targets [10,11].) 3) The stretches of perfect complementarity within microRNA hits in the outlier mRNA set were not biased to occur near the 5'-end of the microRNA. This is not necessarily at odds with earlier analyses, since our outlier set includes only perfect stretches of 13 bases or more, and the 5' end may be more critical in those cases where only a short perfect stretch of complementarity exists.

One might object that our ability to detect certain trends seen in *Drosophila *and *C. elegans *was simply obscured by the fact that we searched the large sequence space represented by all human mRNA sequences – the larger the sequence space, the greater the chance that any given target criterion will be satisfied by scrambled sequences, hence the more difficult it can be to detect true targets above the noise level. We agree that this can be a problem using very large sequence databases, such as the human EST database or the entire human genome. As well, using cut-off levels of parameter distributions to define the candidate list probably excludes many true human mRNA targets. However, human RefSeq was demonstrably not too large for our analysis, since very strong trends were observed in a variety of other parameters (figs. 1,2,3,4).

Based upon sequence complementarity, at least 57 out of the 71 members of the outlier set are predicted to represent true microRNA targets (Table 1). Indeed, since this paper was first submitted for publication, one of the mRNAs on this list, HOXB8, has been experimentally confirmed [24]. Note, however, that accessory factors in the RISC might also help to determine which potential mRNA targets will actually be sites of regulation in vivo. As well, microRNA and target must be expressed in the same times and places in adequate concentrations; secondary structure of the mRNA target region may be important [19,20]; see also [8]; and RNA A-to-I editing [25,26] might operate to prevent certain target sequences from binding microRNAs adequately.

## Conclusions

In summary, the population-wide characteristics of microRNA-mRNA sequence complementarity indicate that microRNAs recognize a subset of human mRNA sequences better than expected by chance. This outlier set does obey a number of properties expected for true biological mRNA targets, but does not show a bias for target regions to be located within the 3'-UTR of the mRNA, and stretches of perfect complementarity are not biased towards the 5'-end of the microRNA. If the candidate list is representative of the full set of biologically significant targets, then the total number of mRNA targets in humans may be much greater than previously proposed [8].

## Abbreviations

5'-UTR, 5'-untranslated region. CDS, protein coding region. 3'-UTR, 3'-untranslated region.

## Methods

### MicroRNAs

Statistical analyses were first carried out using the set of mouse and human microRNAs listed in Lagos-Quintana et al [15], and then repeated to obtain individual candidate mRNA targets using all human microRNAs listed on the Sanger microRNA repository [16] as of December 2003. These sources were combined to create nonredundant microRNA sets (i.e. microRNAs that have 10 or more consecutive nucleotides in common were collected into groups and the longest member of the group was chosen as nonredundant). Almost all mouse microRNAs have exact human counterparts, but hits were annotated with mouse entries in cases of minor corrections and discrepancies between these two sources. One individual microRNA (mir-207) and several scrambled sequences were found to be low-complexity or complementary to abundant repeats (e.g., Alu) and were removed from consideration.

### mRNAs

Analyses were first carried out using the set of human RefSeq mRNAs available in August 2003, and then supplemented with additional human RefSeq mRNAs listed as of December 2003. A) Sequences in RefSeq > 20,000 bases long were removed from consideration because they were hit by many, if not all microRNAs, and a few sequences > 15,000 bases long were removed from the final candidate list because they had a relatively high false-positive probability. B) When counting the number of hits over the population of mRNAs, two hits were counted as redundant if the entire region around the hit (plus or minus 25 nucleotides on each side) was identical. C) When counting distinct hits by microRNAs on the same target, two hits were counted as redundant if they shared the same exact hit. This minimized possible artifacts due to overlapping microRNAs, as well as removed cases in which microRNAs hit exactly-repeating sequences within the target. D) In tabulating hits onto mRNA targets, we did not count hits that contained low-complexity sequences as detected by the DUST algorithm encoded by a Perl script provided by Lincoln Stein [27]. E) When assembling the candidate mRNA target list, we chose a single exemplary mRNA and removed other entries that were transcript variants or nearly identical by BLAST searching. In the course of this study, some of the target mRNAs were removed from RefSeq for routine genome annotation processing. If these were subsequently replaced with updated versions of these mRNAs in RefSeq that included the same hits, the latter version is listed here as well. For those entries removed but not replaced in RefSeq at the time of submission of the manuscript, other active entries currently in Genbank are listed if possible.

### Statistics

To decide whether the number of observed microRNA hits were significantly different from chance, 10 replications of scrambled sequences were used to estimate prediction intervals. The prediction interval allows one to say with 95% confidence that any single new replication of the scrambled set will be below the value of the microRNA set. Prediction intervals were chosen as more conservative and more appropriate than confidence intervals.

## Authors' contributions

NS contributed biological expertise, whereas VT contributed statistical and computational expertise. The analyses were carried out together, and both authors read and approved the final manuscript.

## Figures and Tables

**Table 1:** The Outlier mRNA Set (Candidate Target List)

Transcription factors and other nucleic-acid binding proteins (15)
homeo box B8 (HOXB8)
E2F transcription factor 6 (E2F6)
transcription factor 20 (AR1) (TCF20)
DEAD (Asp-Glu-Ala-Asp) box polypeptide 51 (DDX51)
similar to ATP-dependent RNA helicase DDX24 (DEAD-box protein 24) (LOC221311)
myeloid/lymphoid or mixed-lineage leukemia (trithorax homolog); translocated to, 1 (MLLT1)
high mobility group AT-hook 2 (HMGA2)
polymerase (DNA directed), theta (POLQ)
strand-exchange protein 1 (SEP1)
hypothetical protein FLJ12994 – RFX DNA-binding domain
similar to LINE-1 reverse transcriptase homolog (LOC285907)
similar to hypothetical protein (L1H 3 region) – related to reverse transcriptase
similar to putative p150 (LOC282945) – related to reverse transcriptase
similar to reverse transcriptase related protein (LOC222252)
similar to RTl1 (LOC376283) – related to reverse transcriptase
Kinases, receptors and other signaling proteins (13)

fyn-related kinase (FRK)
WNK kinase, lysine deficient 3 (PRKWNK3)
protein phosphatase 2, regulatory subunit B (B56), epsilon isoform (PPP2R5E)
EphA5 receptor (EPHA5)
killer cell lectin-like receptor subfamily A, member 1 (KLRA1)
polycystin and REJ (sperm receptor for egg jelly homolog, sea urchin)-like (PKDREJ)
integrin, alpha X (antigen CD11C (p150), alpha polypeptide) (ITGAX)
inositol 1,4,5-triphosphate receptor, type 1 (ITPR1)
hypothetical protein FLJ32810 – RhoGAP domain, SH3 domain
hypothetical protein FLJ00058 – G protein gamma 7
Rho GTPase-activating protein (RICS)
hypothetical protein FLJ30899 – probable ras GAP
similar to ADP-ribosylation factor-like membrane-associated protein (LOC132946) – ARF-like small GTPase domain, Sar1p-like member of the Ras-family
Membrane and extracellular proteins (11)

Laminin, beta 4 (LAMB4)
laminin, gamma 2 (LAMC2)
fibronectin 1 (FN1)
collagen, type IV, alpha 5 (Alport syndrome) (COL4A5)
collagen, type XIX, alpha 1 (COL19A1)
similar to Voltage-dependent anion-selective channel protein 1 (VDAC-1)
ATPase, Na+/K+ transporting, alpha 2 (+) polypeptide (ATP1A2)
complement component 1, q subcomponent, beta polypeptide (C1QB)
hypothetical protein FLJ20506 – transmembrane protein
MAM domain containing glycosylphosphatidylinositol anchor 1 (MDGA1) – Ig, MAM domains
similar to TCAM-1 (LOC284171)
Cytoskeletal domain-containing proteins (7)

myosin heavy chain Myr 8 (MYR8)
ankyrin repeat domain 17 (ANKRD17)
KIAA1817 protein – intermediate filament, ATPase, PDZ, Band 4.1, FERM domains
chromosome 10 open reading frame 39 (C10orf39) – homologous to myosin, plectin
oxysterol binding protein 2 (OSBP2) – pleckstrin homology domain
KIAA1202 protein – PDZ, ATPase domains
hypothetical protein FLJ23529 – homolgous to dynein heavy chain
Miscellaneous or unknown function (26)

cell cycle progression 2 protein (CPR2)
olfactomedin 3 (OLFM3)
histidine rich calcium binding protein (HRC)
interferon-related developmental regulator 1 (IFRD1)
KIAA1301 protein – NEDD4-related E3 ubiquitin ligase NEDL2
KIAA1203 protein – ubiquitin C-terminal hydrolase
hydroxyprostaglandin dehydrogenase 15-(NAD) (HPGD)
UDP-GlcNAc:betaGal beta-1,3-N-acetylglucosaminyltransferase 1 (B3GNT1)
KIAA1854 protein – leucine rich repeat C-terminal domains
testis specific, 14 (TSGA14)
chromosome 4 open reading frame 1 (C4orf1)-membrane AND nuclear protein
hypothetical protein FLJ33069
hypothetical protein FLJ38464
hypothetical protein LOC285431
hypothetical protein LOC284107
similar to agCP1362 [Anopheles gambiae str. PEST] (LOC344751)
KIAA1632 protein
similar to hypothetical protein D11Ertd497e (LOC343360)
LOC138724
LOC343460
LOC340963
LOC343220
LOC285842
LOC352767
LOC350293

## References

[B1] Lai EC (2003). microRNAs: runts of the genome assert themselves. Curr Biol.

[B2] Carrington JC, Ambros V (2003). Role of microRNAs in plant and animal development. Science.

[B3] Nelson PT, Hatzigeorgiou AG, Mourelatos Z (2004). miRNP:mRNA association in polyribosomes in a human neuronal cell line. RNA.

[B4] Sempere LF, Freemantle S, Pitha-Rowe I, Moss E, Dmitrovsky E, Ambros V (2004). Expression profiling of mammalian microRNAs uncovers a subset of brain-expressed microRNAs with possible roles in murine and human neuronal differentiation. Genome Biol.

[B5] Enright AJ, John B, Gaul U, Tuschl T, Sander C, Marks DS (2003). MicroRNA targets in Drosophila. Genome Biol.

[B6] Stark A, Brennecke J, Russell RB, Cohen SM (2003). Identification of Drosophila MicroRNA Targets. PLOS Biol.

[B7] Rajewsky N, Socci ND Computational identification of microRNA targets. Dev Biol.

[B8] Lewis BP, Shih I-h, Jones-Rhoades MW, Bartel DP, Burge CB (2003). Prediction of Mammalian MicroRNA Targets. Cell.

[B9] Kiriakidou M, Nelson PT, Kouranov A, Fitziev P, Bouyioukos C, Mourelatos Z, Hatzigeorgiou A (2004). A combined computational-experimental approach predicts human microRNA targets. Genes Dev.

[B10] Llave C, Xie Z, Kasschau KD, Carrington JC (2002). Cleavage of Scarecrow-like mRNA targets directed by a class of Arabidopsis miRNA. Science.

[B11] Rhoades MW, Reinhart BJ, Lim LP, Burge CB, Bartel B, Bartel DP (2002). Prediction of plant microRNA targets. Cell.

[B12] Lim LP, Lau NC, Weinstein EG, Abdelhakim A, Yekta S, Rhoades MW, Burge CB, Bartel DP (2003). The microRNAs of Caenorhabditis elegans. Genes Dev.

[B13] Aravin AA, Lagos-Quintana M, Yalcin A, Zavolan M, Marks D, Snyder B, Gaasterland T, Meyer J, Tuschl T (2003). The small RNA profile during Drosophila melanogaster development. Dev Cell.

[B14] Lai EC (2002). Micro RNAs are complementary to 3' UTR sequence motifs that mediate negative post-transcriptional regulation. Nat Genet.

[B15] Lagos-Quintana M, Rauhut R, Yalcin A, Meyer J, Lendeckel W, Tuschl T (2002). Identification of tissue-specific microRNAs from mouse. Curr Biol.

[B16] The miRNA Registry. http://www.sanger.ac.uk/Software/Rfam/mirna/.

[B17] RefSeq. http://www.ncbi.nlm.nih.gov/RefSeq/.

[B18] Pusch O, Boden D, Silbermann R, Lee F, Tucker L, Ramratnam B (2003). Nucleotide sequence homology requirements of HIV-1-specific short hairpin RNA. Nucleic Acids Res.

[B19] Kretschmer-Kazemi Far R, Sczakiel G (2003). The activity of siRNA in mammalian cells is related to structural target accessibility: a comparison with antisense oligonucleotides. Nucleic Acids Res.

[B20] Bohula EA, Salisbury AJ, Sohail M, Playford MP, Riedemann J, Southern EM, Macaulay VM (2003). The efficacy of small interfering RNAs targeted to the type 1 insulin-like growth factor receptor (IGF1R) is influenced by secondary structure in the IGF1R transcript. J Biol Chem.

[B21] Varani G, McClain WH (2000). The G × U wobble base pair. A fundamental building block of RNA structure crucial to RNA function in diverse biological systems. EMBO Rep.

[B22] Altschul SF, Madden TL, Schaffer AA, Zhang J, Zhang Z, Miller W, Lipman DJ (1997). Gapped BLAST and PSI-BLAST: a new generation of protein database search programs. Nucleic Acids Res.

[B23] Michael MZ, O' Connor SM, van Holst Pellekaan NG, Young GP, James RJ (2003). Reduced accumulation of specific microRNAs in colorectal neoplasia. Mol Cancer Res.

[B24] Yekta S, Shih IH, Bartel DP (2004). MicroRNA-directed cleavage of HOXB8 mRNA. Science.

[B25] Scadden AD, Smith CW (2001). RNAi is antagonized by A-->I hyper-editing. EMBO Rep.

[B26] Tonkin LA, Bass BL (2003). Mutations in RNAi rescue aberrant chemotaxis of ADAR mutants. Science.

[B27] Bioperl: Repetitive DNA. http://bioperl.org/pipermail/bioperl-l/1999-November/003313.html.

